# Long-Term Effects of Tolvaptan Therapy on Total Kidney Volume and Renal Function in Patients with Autosomal Dominant Polycystic Kidney Disease: A Single-Center Experience

**DOI:** 10.3390/jcm14186537

**Published:** 2025-09-17

**Authors:** Vassilis Filiopoulos, Ioannis Kofotolios, Kalliopi Vallianou, Efstratios Karavasilis, Georgios Ntounas, Christina Melexopoulou, Smaragdi Marinaki

**Affiliations:** 1 Clinic of Nephrology and Renal Transplantation, Laiko General Hospital, Medical School of Athens, National and Kapodistrian University, 11527 Athens, Greecekallia_harry@hotmail.com (K.V.); xmelexopoulou@gmail.com (C.M.);; 2 Medical Physics Laboratory, Democritus University of Thrace, 69100 Alexandroupolis, Greece; 3 Sismanogleio General Hospital, 15126 Athens, Greece; 4 Iatrodiagnosi Diagnostic Medical Centers, 14122 Athens, Greece

**Keywords:** tolvaptan, autosomal dominant polycystic kidney disease, TKV growth

## Abstract

**Background:** Tolvaptan, a vasopressin V2 receptor antagonist, is the only approved disease-modifying therapy for Autosomal Dominant Polycystic Kidney Disease (ADPKD), yet real-world data on its long-term effectiveness remain limited. **Methods:** In this single-center retrospective study, we evaluated 30 patients with ADPKD who received tolvaptan therapy for at least three years between 2019 and 2024. All patients met standard inclusion criteria and underwent serial magnetic resonance imaging to assess total kidney volume (TKV), along with longitudinal monitoring of renal function using estimated glomerular filtration rate (eGFR). **Results:** At the end of follow-up, the median annual TKV growth rate was 4.27% (IQR: 1.39–7.98), which did not differ significantly from the predicted without treatment growth rate of 5.3% (95% CI: −2.75 to 0.69, *p* = 0.194). Although the impact on TKV was limited, tolvaptan notably slowed the decline in kidney function, with a median eGFR of 65 mL/min/1.73 m^2^ at follow-up, compared to a predicted value of 60.8 mL/min/1.73 m^2^ (95% CI: −14.60 to −6.18, *p* < 0.001), reflecting a 33.9% relative benefit. In 80% of patients, renal function after three years was better than predicted. **Conclusions:** These findings suggest that tolvaptan provides significant functional benefit in ADPKD patients in routine clinical practice, even in the absence of marked suppression in TKV growth and support its continued use in carefully selected individuals.

## 1. Introduction

ADPKD is a prevalent hereditary renal disorder, and it accounts for approximately 5–10% of end-stage kidney disease (ESKD) cases worldwide [1]. ADPKD is marked by the gradual formation of numerous fluid-filled cysts in the kidneys, which leads to an increase in TKV, hypertension, and eventually loss of kidney function [2]. In ADPKD, cyst growth is fueled by excessive cell proliferation and the accumulation of fluid within the cyst cavity, both of which are influenced by signaling pathways such as cyclic adenosine monophosphate (cAMP) [3]. Tolvaptan, a vasopressin V2 receptor antagonist, is the first approved treatment shown to modify the course of ADPKD by interfering with a central pathway responsible for cyst expansion [4,5]. By inhibiting V2 receptors it leads to decreased cAMP levels—subsequently suppressing epithelial cell growth and fluid accumulation within cysts and thereby slowing the increase in TKV [6]. Evaluating TKV is essential for predicting the progression of ADPKD. A range of studies, notably the pivotal CRISP trial, have validated TKV as a reliable imaging indicator that correlates with the extent of structural kidney damage and aids in categorizing patients based on their likelihood of rapid disease advancement [7,8,9]. Regular monitoring of TKV allows clinicians to pinpoint individuals at greater risk, facilitating early treatment decisions and enabling more personalized approaches to managing the disease. Previous clinical trials have provided compelling evidence for the efficacy and safety of tolvaptan in ADPKD patients. In particular the TEMPO 3:4 trial demonstrated that tolvaptan significantly reduced the rate of TKV growth and slowed the decline in eGFR over three years, establishing it as the first disease-modifying therapy for ADPKD [4]. The subsequent TEMPO 4:4 extension suggested that the early benefits on kidney function could be sustained with long-term treatment, although suppression of TKV growth was less consistent [10]. Beyond randomized controlled trials, real-world studies have provided more heterogeneous results. Observational cohorts have generally confirmed the slowing of eGFR decline, with relative treatment benefits compared to predicted progression [11,12]. However, the effect on TKV growth has been less robust outside trial settings, with several studies reporting non-significant or modest changes [13,14]. Consequently, real-world data and single-center experiences are essential to validate and complement trial findings, offering valuable perspectives on patient eligibility, long-term adherence and treatment outcomes across more varied and representative populations [15]. This study aims to evaluate the long-term impact of tolvaptan therapy on TKV progression in a cohort of ADPKD patients managed at our center, through an analysis of imaging and clinical data collected over a three-year follow-up period.

## 2. Materials and Methods

### 2.1. Study Population

This is a single-center cohort study of patients with ADPKD, who received tolvaptan between 2019 and 2024. Eligibility criteria for tolvaptan treatment included age between 18 and 55 years, an eGFR greater than 25 mL/min/1.73 m^2^, and a Mayo Clinic Imaging Classification (MCIC) [16] of 1C, 1D, or 1E. All patients enrolled in the study received tolvaptan for a minimum of three years and underwent at least two magnetic resonance imaging (MRI) scans—one prior to treatment initiation and another at least three years after starting treatment. Patients who discontinued tolvaptan for adverse events (thirst, polyuria, hepatotoxicity and family planning) or were exposed to treatment for less than 3 years were excluded from the study.

### 2.2. Clinical and Laboratory Data

We recorded patients’ demographic (age, sex, MBI), clinical characteristics and medication use including renoprotective agents. A detailed biochemical assessment was conducted monthly during the initial 18 months, followed by evaluations every three months thereafter. The laboratory parameters included a complete blood count, serum creatinine and urea levels, c-reactive protein, electrolytes, liver function tests and urinalysis. Renal function and disease progression were assessed using eGFR. Renal function and disease progression were assessed using eGFR, Regarding the baseline and observed eGFR the CKD-EPI 2009 equation was used [17]. The predicted eGFR without tolvaptan treatment was estimated using the Mayo classification formula [18]. The Mayo classification is a widely used imaging-based prognostic tool for patients with ADPKD. The classification relies on height-adjusted total kidney volume (htTKV) measured by MRI or CT, adjusted for age, and categorizes patients into five subclasses (1A–1E) for typical ADPKD. Validation studies across multiple cohorts have confirmed its ability to predict renal function decline more accurately than eGFR alone [19,20]. In addition, TKV was measured using an MRI stereological semiautomatic method and was compared to the predicted annual TKV increase of 5.3% over three years in the absence of tolvaptan treatment [8,21].

### 2.3. Outcome Measurements

We defined the primary outcome as the long-term impact of tolvaptan on TKV progression, and the secondary outcome as renal progression after ≥3 years of therapy.

### 2.4. Statistical Analysis

Descriptive statistics are presented as mean ± standard deviation for normally distributed continuous variables, and as median with interquartile range for skewed continuous variables. Normality of distribution was assessed using the Shapiro–Wilk test. Group differences were analyzed using the independent samples *t*-test for normally distributed continuous variables, and the Mann–Whitney U test for non-normally distributed variables. Categorical variables were compared using the chi-square test. Comparisons between predicted and observed values of eGFR and TKV were performed using the paired *t*-test or the Wilcoxon signed-rank test, as appropriate. All statistical analyses were conducted using Stata software (version 14.2; StataCorp LLC, College Station, TX, USA). A *p*-value < 0.05 was considered statistically significant.

## 3. Results

### 3.1. Study Cohort

**Figure 1:**
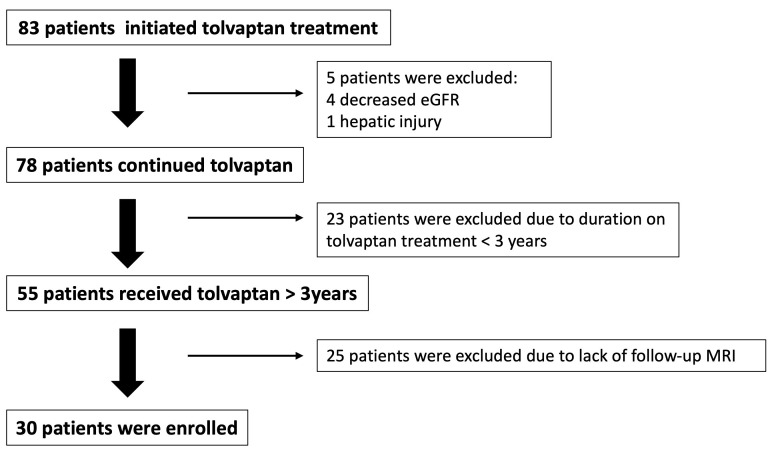
Study flow diagram illustrating patient selection and inclusion. eGFR: estimated Glomerular Filtration Rate, MRI: Magnetic Resonance Imaging.

A total of 83 patients, who met the aforementioned inclusion criteria, received tolvaptan treatment at our center during the study period from 2019 to 2024. Of these, 53 were excluded from the primary analysis: 5 patients due to adverse effects—4 due to renal impairment and 1 due to hepatotoxicity, characterized by greater than 5-fold increase in transaminase levels. In addition, 23 patients had <3 years of exposure, and 25 lacked a ≥3-year follow-up MRI (mainly due to missed imaging appointments despite ongoing clinical and laboratory follow-up). Ultimately, 30 patients were enrolled in the study (Figure 1).

### 3.2. Baseline Patient Characteristics

Table 1 provides an overview of the baseline demographic and clinical features of the study cohort. Among the 30 patients included, the majority were female (56.6%), with a median age of 41 years (interquartile range [IQR]: 35–47 years). A positive family history of ADPKD was identified in 93.3% of the patient population. The majority of patients (28/30, 93.3%) received a renin–angiotensin–aldosterone system (RAAS) inhibitor during the study period, while no other kidney-protective agents such as sodium–glucose cotransporter 2 inhibitors (SGLT2i), finerenone, or glucagon-like peptide-1 receptor agonists (GLP-1 RA) were initiated throughout follow-up period. According to the MCIC, the distribution at baseline was as follows: class 1C (*n* = 13, 43.3%), class 1D (*n* = 14, 46.6%), and class 1E (*n* = 3, 10.0%).

### 3.3. Tolvaptan Dosing

The initial tolvaptan dose was 60 mg/day, with gradual titration up to a maximum of 120 mg/day based on patient tolerance. During the early phase of the treatment, 24 h urine osmolality was used to guide tolvaptan dose adjustment; however, this approach was subsequently abandoned and urine osmolality <250 mOsm/kg was thereafter employed to assess treatment adherence. Because dose levels frequently fluctuated over time, a single ‘maximum tolerable dose’ for the entire 3-year period is not meaningful. Instead, we report the median daily dose at the end of follow-up and (120 mg/day). A total of 18 patients (60%) received the maximum dose of 120 mg/day, 9 patients (30%) remained on the lowest dose (60 mg/day), and the rest maintained an intermediate dose (90 mg/day).

### 3.4. Tolvaptan Effects on TKV Growth

**Figure 2:**
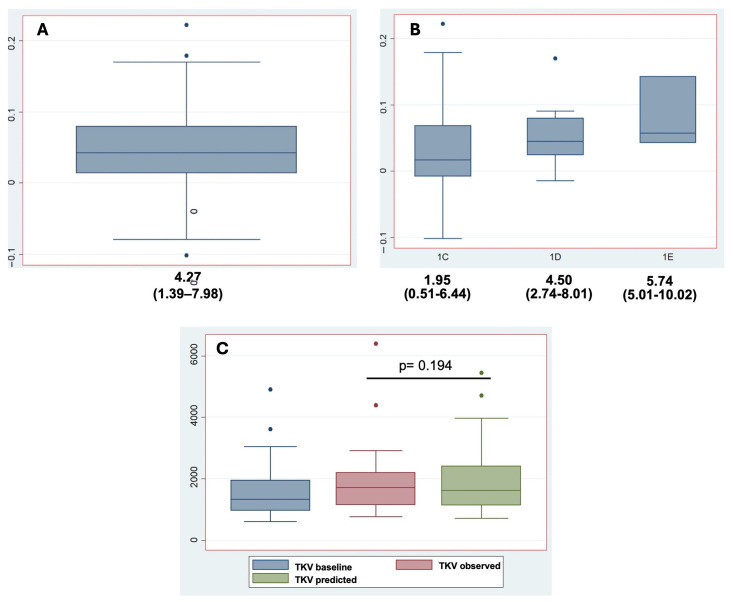
Effect of tolvaptan therapy on TKV in patients with ADPKD. (**A**) Distribution of annualized TKV growth rate (%) for the overall study cohort. (**B**) Stratified analysis of annualized TKV growth rate by MCIC. (**C**) Comparison between baseline TKV, predicted TKV without treatment, and observed TKV following tolvaptan therapy. A non-significant difference was observed between predicted and observed TKV at follow-up (95% CI: −2.75 to 0.69, *p* = 0.194). Box plots represent median values with IQR. TKV: Total Kidney Volume, MCIC: Mayo Clinic Imaging Classification, IQR: interquartile range.

There was no statistically significant difference in TKV before and three years after initiation of chronic tolvaptan treatment: median TKV increased from 1323 mL (IQR: 961–1948) to 1704 mL (IQR: 1149–2202), (95% CI: −813.44 to 309.17, *p* = 0.294). Similarly, no significant change in TKV growth was observed when stratified by MCIC category (Table 2). When comparing observed TKV at the end of follow-up with the predicted TKV (based on an estimated annual increase of 5.3%), no significant difference was found: 1704 mL (IQR: 1149–2202) vs. 1617 mL (IQR: 1134–2404), corresponding to an annual growth rate of 4.27% and 5.30%, respectively (95% CI: −2.75 to 0.69, *p* = 0.194) (Figure 2). Interestingly, patients classified as MCIC 1C exhibited a lower TKV growth rate of 1.95% per year.

### 3.5. Tolvaptan Effects on eGFR

**Figure 3:**
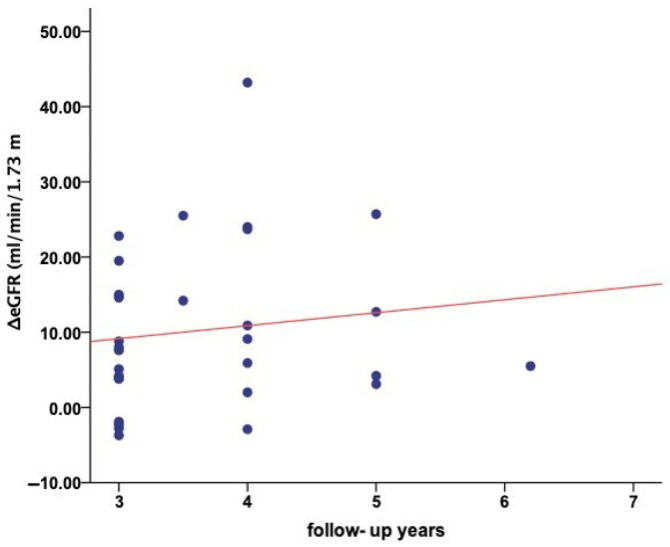
Association between duration of tolvaptan therapy and the difference between observed and predicted eGFR (ΔeGFR). Scatter plot illustrating the relationship between follow-up duration (years) and ΔeGFR, defined as the difference between observed and predicted eGFR values, in patients with ADPKD undergoing tolvaptan therapy. Each dot represents an individual patient. eGFR: estimated glomerular filtration rate, ADPKD: autosomal dominant polycystic kidney disease.

The median (IQR) duration of the period during tolvaptan treatment was 3.5 (3–4) years. The effect of tolvaptan on the decline of eGFR was assessed by comparing the observed eGFR with the predicted eGFR (Table 3). An acute slope in eGFR was observed one month after tolvaptan treatment initiation, with the median (IQR) eGFR declining from 77 (54–97) mL/min/1.73 m^2^ at baseline to 74 (52.5–93.5) mL/min/1.73 m^2^ (95% CI: 0.31 to 6.51, *p* = 0.032). At the end of follow-up, the observed eGFR was 65 (46–100.5) mL/min/1.73 m^2^, significantly lower than baseline values (95% CI: 3.06 to 11.34 *p* = 0.001). The median predicted eGFR at the same time point was 60.8 (38.3–79.3) mL/min/1.73 m^2^. The observed eGFR was significantly higher than the predicted value (5% CI: −14.60 to −6.18, *p* < 0.001) further suggesting that longer follow-up may be associated with better preservation or improvement of renal function (Figure 3). In 80% of patients, the observed eGFR at follow-up was higher than the predicted eGFR.

## 4. Discussion

In this retrospective observational study, we evaluated the long-term effects of tolvaptan in a real-world cohort of patients with ADPKD who were treated for more than three years. Although no statistically significant difference was observed between observed and predicted TKV, the decline in eGFR was significantly slower than the rate predicted using the Mayo classification formula. These findings align with an emerging body of evidence indicating that the therapeutic benefit of tolvaptan on kidney function may occur independently of measurable changes in kidney volume [22,23].

A total of 30 patients met the eligibility criteria and were included in the analysis, with the majority receiving the maximum tolerated dose of tolvaptan. The median annual TKV growth rate in this cohort was 4.27%, which was numerically lower than the predicted annual increase of 5.3%, although the difference did not reach statistical significance. This result contrasts with findings from the TEMPO 3:4 trial and later studies [4,21,24], where tolvaptan was associated with a significant reduction in TKV growth. However, such trials were conducted under controlled conditions with close patient monitoring and adherence support, which may not fully reflect routine clinical practice. Consequently, real-world studies are essential to assess the long-term efficacy of tolvaptan outside of clinical trial settings, though such data remain limited. The open-label extension study TEMPO 4:4 [10] found that the initial reduction in TKV observed during early tolvaptan therapy was not sustained over time, with kidney volumes continuing to increase at a rate like pre-treatment levels. Similar observations have been reported in studies by Higashihara et al. and Zhou et al. [11,12], as well as in more recent real-world cohorts, which also showed no significant attenuation in TKV progression [13,14]. Notably, in our study, patients classified as MCIC 1C exhibited the lowest TKV growth rate (1.95% per year), suggesting that treatment response may vary by disease stage or cyst burden.

Renal function, assessed by eGFR, demonstrated a clearer treatment-related effect compared to TKV. An initial, statistically significant decline in eGFR was observed one month after tolvaptan initiation, consistent with the known acute hemodynamic response to tolvaptan [5,25,26]. Over the three-year treatment period, the median decline in observed eGFR was −2.66 mL/min/1.73 m^2^/year, whereas the predicted decline based on the Mayo classification formula was −4.03 mL/min/1.73 m^2^/year, corresponding to a relative treatment effect of 33.9%. This magnitude of functional benefit is comparable to results from major clinical trials, including TEMPO 3:4 (−2.72 vs. −3.70 mL/min/1.73 m^2^/year) and REPRISE (−2.34 vs. −3.61 mL/min/1.73 m^2^/year) [4,5], where tolvaptan significantly slowed eGFR decline compared to placebo. Similarly, in real-world studies, Edwards et al. [27] reported a 37.1% reduction in eGFR slope among 97 patients, and Zhou et al. [12] found a 26.4% benefit in a pooled analysis of 1186 patients from several interventional studies. Longer-term observational data have shown variable results; Yamazaki et al. reported a 40% treatment effect over six years in 55 patients, whereas Higashihara et al. observed a 15.2% benefit, and Geertsema et al. reported an improvement of 14.1% [11,14,24]. These differences may reflect variations in patient selection, baseline disease severity, treatment adherence, and follow-up duration across studies.

In our cohort, tolvaptan provided a clear benefit on eGFR trajectory despite only modest effects on TKV, consistent with prior real-world studies and randomized controlled trials demonstrating the efficacy of tolvaptan in slowing the decline of kidney function [4,10,13,14,24]. The novelty of our findings lies in confirming that meaningful preservation of kidney function can occur independently of significant TKV suppression. This perspective aligns with the broader shift in kidney care from purely anatomical or volumetric endpoints toward function preservation as the primary therapeutic goal. A parallel can be drawn from renal oncology, where nephron-sparing strategies and predictive tools such as RENSAFE [28] emphasize long-term renal function over purely anatomical measures. This dissociation between structural and functional outcomes may reflect the multifactorial nature of ADPKD pathophysiology. Although cyst expansion is a central driver of disease, multiple parallel processes influence long-term renal function. Histopathologic and imaging studies highlight the roles of tubular atrophy, interstitial fibrosis, inflammation, and dysregulated renal hemodynamics as key drivers of disease progression. Together, these mechanisms provide a plausible explanation for why therapies such as tolvaptan can yield measurable functional benefit even when effects on global kidney volume are modest. Translationally, these observations argue for reorienting ADPKD research and clinical endpoints toward function preservation. In parallel, new prognostic scores and imaging biomarkers that better reflect parenchymal health, such as automated cyst segmentation metrics or parenchymal fraction, may complement or surpass reliance on TKV alone offering a more precise framework for risk stratification and treatment selection.

Our study has certain limitations. The small sample size (*n* = 30) limits generalizability of the results. Since over half of the initial cohort was excluded, the study population may be biased toward individuals who tolerated therapy and successfully completed imaging. Volumetric imaging was performed only at baseline and at the end of follow-up, restricting assessment of short-term TKV changes. Moreover, the lack of genotype information and unmeasured confounding variables—such as blood pressure control, glycemic status, and medication adherence—may have influenced eGFR trajectories and introduced bias into the functional outcome analysis. Although the Mayo eGFR-prediction equation has external validation in multiple cohorts, it has not been specifically calibrated for Greek populations and may underestimate decline in MIC 1C–1E; therefore, comparisons between observed and predicted eGFR should be interpreted cautiously.

## 5. Conclusions

In conclusion, our findings contribute to the growing body of real-world evidence supporting the long-term functional effect of tolvaptan in ADPKD. The slower-than-predicted eGFR decline observed in the majority of patients, even in the absence of significant TKV suppression, underscores the clinical benefit of treatment and highlights the importance of individualized disease monitoring in this heterogeneous population.

## Figures and Tables

**Table 1:** Baseline demographic and clinical characteristics of the study population.

	*n* = 30
Female, *n* (%)	17 (56.7)
Age, years, median (IQR)	35 (29.5–44.5)
Body weight, kg, median (IQR)	77.2 (57.5–87.5)
Height, m, median (IQR)	1.75 (1.67–1.82)
BMI, kg/m^2^, median (IQR)	24.8 (21.1–26.9)
Family Hx of ADPKD, *n* (%)	27 (90)
Hypertension, *n* (%)	18 (60)
Smoking, *n* (%)	11 (36.7)
eGFR mL/min/1.73 m^2^ (at tolvaptan initiation), median (IQR)	77 (54–97)
TKV, mL (at tolvaptan initiation), median (IQR)	1323 (940–1956)
MAYO classification *n*, (%)	
1C	13 (43.3)
1D	14 (46.7)
1E	3 (10)

ADPKD: Autosomal Polycystic kidney Disease, BMI: Body Mass Index TKV: Total Kidney Volume, eGFR: estimated Glomerular Filtration Rate.

**Table 2:** Baseline and follow-up TKV and TKV growth rate according to MCIC.

	All (*n* = 30)	1C (*n* = 13)	1D (*n* = 14)	1E (*n* = 3)
TKV baseline, mL, median (IQR)	1323 (961–1948)	1293 (982–1570)	1565 (921–2163)	1469 (949–4894)
Follow-up, years, median (IQR)	3.5 (3–4)	3.25 (3–4)	3.75 (3–4)	4 (3–5)
TKV observed, mL, median (IQR)	1704 (1149–2202)	1270 (956–1767)	1810 (1490–2829)	1811 (1149–6394)
TKV growth rate *, %/year, median (IQR)	4.27 (1.39–7.98)	1.95 (0.51–6.44)	4.50 (2.74–8.01)	5.74 (5.01–10.02)

TKV: Total kidney volume, IQR: interquartile range, MCIC: Mayo Clinic Imaging Classification * TKV growth rate was measured as the median difference in TKV divided by the follow-up time in years.

**Table 3:** Comparison of baseline, predicted, and observed eGFR by MCIC.

	eGFR Baseline, mL/min/1.73 m^2^, Median (IQR)	Predicted eGFR, mL/min/1.73 m^2^, Median (IQR)	Observed eGFR mL/min/1.73 m^2^, Median (IQR)	*p*-Value *	95% CI
All (*n* = 30)	77 (54–97)	60.8 (38.3–79.3)	65 (46–105.5)	<0.001	−14.60 to −6.18
1C (*n* = 13)	65 (49.5–82)	48.5 (41.1–70)	62 (45.7–88)	0.004	−15.60 to −3.69
1D (*n* = 14)	84 (56.2–93.7)	60 (39.6–84.3)	64.05 (48.2–90.2)	0.008	−12.29 to −2.23
1E (*n* = 3)	98 (72–109)	61 (45.9–72.5)	104 (75–107)	0.001	−63.33 to −7.39

* *p* value calculated between predicted and observed eGFR. eGFR: estimated glomerular filtration rate, IQR: interquartile ranges. MCIC: Mayo Clinic Imaging Classification.

## Data Availability

Data are available upon request.

## Abbreviations

The following abbreviations are used in this manuscript:

ADPKD	Autosomal Dominant Polycystic Kidney Disease
TKV	Total Kidney Volume
IQR	Interquartile Ranges
eGFR	estimated Glomerular Filtration Rate
ESKD	End-Stage Kidney Disease
cAMP	Cyclic Adenosine Monophosphate
MCIC	Mayo Clinic Imaging Classification
MRI	Magnetic Resonance Imaging
BMI	Body Mass Index
